# The first new species of European *Ascocotyle* Looss, 1899 (Digenea: Heterophyidae) described in more than half a century

**DOI:** 10.1016/j.ijppaw.2021.10.003

**Published:** 2021-10-11

**Authors:** Andrea Gustinelli, Monica Caffara, Dino Scaravelli, Maria Letizia Fioravanti, Tomáš Scholz

**Affiliations:** a Department of Veterinary Medical Sciences, Alma Mater Studiorum, University of Bologna, via Tolara di Sopra 50, 40064, Ozzano Emilia (BO), Italy; b Institute of Parasitology, Biology Centre of the Czech Academy of Sciences, Branišovská 1160/31, 370 05, České Budějovice, Czech Republic

**Keywords:** Trematoda, *Ascocotyle trentinii* n. sp., Taxonomy, 28S rDNA, *Aphanius fasciatus*, Brackish water, Italy

## Abstract

*Ascocotyle* (*Phagicola*) *trentinii* n. sp. is described based on adults from experimentally infected ducklings (*Anas platyrhynchos domesticus*) fed with metacercariae from the visceral serosa of the Mediterranean banded killifish, *Aphanius fasciatus* (Cyprinodontiformes: Aphaniidae), from coastal lagoons in northeastern Italy (Emilia-Romagna Region). The new species is placed into the subgenus *Phagicola* because of the presence of a single row of circumoral spines, vitelline follicles being confined between the ventral sucker and testes, and uterine loops not reaching anterior to the ventral sucker. *Ascocotyle* (*P.*) *trentinii* n. sp. differs from other members of the subgenus *Phagicola*, as well as other species of *Ascocotyle*, by the number (27–33) of circumoral spines which are 13.5–17 μm long and 3.5–5 μm wide, and by the morphology of a gonotyl which is composed of about 8 large refractile pockets. The occurrence of metacercariae in *A. fasciatus* indicates that the life cycle of the new species is completed in brackish water lagoons. It is the fourth species of *Ascocotyle* described in Europe and may be endemic to the Mediterranean region because its second (fish) intermediate host is endemic to this region.

## Highlights

• A new species of *Ascocotyle* (Digenea: Heterophyidae) is described from Europe after more than half a century.
• The new species is characterised by a unique number (27–33) of circumoral spines.
• Mediterranean banded killifish,*Aphanius fasciatus*(Cyprinodontiformes), from coastal lagoons is a second intermediate host.
• *Ascocotyle pindoramensis*from South America, which has no circumoral spines, is most closely related to the new species.

## Graphical abstract

**Figure:**
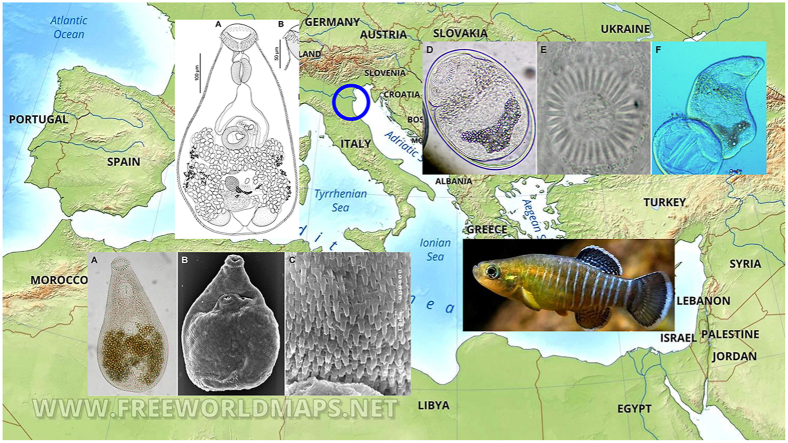

## 1 Introduction

Trematodes of the family Heterophyidae Leiper, 1909, which comprises 36 genera, are mainly parasites of fish-eating birds and mammals (Pearson, 2008). Some of them infect humans, especially in South East Asia (Chai and Jung, 2017). They use prosobranchiate snails as first and fish as second intermediate hosts; fish-eating birds or mammals serve as definitive hosts (Cribb et al., 2003). Some species of the genera *Haplorchis* Looss, 1899, *Heterophyes* Cobbold, 1886, *Metagonimus* Katsurada, 1912, *Procerovum* Onji & Nishio, 1916, *Stellantchamus* Onji & Nishio, 1916, *Centrocestus* Looss, 1899, etc., are causative agents of human fish-borne parasitic disease, with the highest number of human cases reported from South East Asia (Chai et al., 2007; Chai and Jung, 2017). A single species of another genus, *Ascocotyle longa* Ransom, 1920, whose metacercariae occur in mullets (Mugilidae), has also been reported as a potential human parasite, with most human cases reported from Brazil (Chieffi et al., 1992; Simões et al., 2010).

The genus *Ascocotyle* includes species characterized by an oral sucker armed with circumoral spines arranged usually in one or two rows (spines are absent in a very few taxa), and bearing a conical muscular prolongation (posterior appendage) of the oral sucker (Pearson, 2008). Spination patterns, i.e., the number of rows of circumoral spines, their number and size, together with some characteristics of internal organs, such as the posterior extent and shape of the intestinal caeca, morphology of the gonotyl, position and extent of vitelline follicles, etc. serve as a basis for species identification (Burton, 1958; Scholz et al., 1997a, b). Several subgenera of *Ascocotyle* have been proposed, but their validity, circumscription and phylogenetic relationships remain unclear, partly because molecular data are available for only a few species (Hernández-Orts et al., 2019; Santos and Borges, 2020).

During a study aimed at evaluating the health status and sexual development of the natural population of the Mediterranean banded killifish, *Aphanius fasciatus* (Valenciennes) (Cyprinodontiformes: Aphaniidae), the fish were found heavily infected with metacercariae of heterophyid trematodes. The metacercariae found in *A. fasciatus* were fed to ducklings (*Anas platyrhynchos domesticus*) to obtain sexually mature adults. Morphological observation revealed their affiliation to *Ascocotyle* because they possess a single row of circumoral spines, a conical muscular prolongation (posterior appendage) of the oral sucker, a gonotyl with refractile bodies and other characteristics typical of this genus (Pearson, 2008). Comparison of these trematodes with other species of *Ascocotyle* has revealed they belong to a new, unknown species, which is described based on morphology and molecular analysis in the present paper. It is the first new species of this heterophyid genus described from Europe after more than a half-century, i.e., since *Ascocotyle septentrionalis* van den Broek, 1967 was described from the harbour seal, *Phoca vitulina* Linnaeus, off the Netherlands (van den Broek, 1967).

## 2 Materials and methods

### 2.1 Specimen collection

Thirty-five *Aphanius fasciatus* were sampled by electrofishing from lagoons and salt pans of the Cervia salt marshes (44°15′15.17″N; 12°20′02.57″E) on the Adriatic coast of Emilia-Romagna Region, northern Italy. Metacercariae were embedded within the serosa of visceral organs (peritoneum). Previous analyses on different batches of *A. fasciatus* sampled from the same area showed high prevalence and high intensity (always more than 100 metacercariae/fish) belonging to the same species on the basis of the number of circumoral spines (see Table 1). Metacercariae were rinsed in saline, observed live under dissecting microscope and then fixed in 70% ethanol for further studies.

**Table 1:** Number of circumoral spines in species of *Ascocotyle*Looss, 1899 (new species in bold).

Species^a^	Distribution^b^	N° spines	Reference
**Species with a single complete circle of circumoral spines**			
*A. ascolonga*Witenberg, 1929	Egypt*, Israel	16	Witenberg (1929), Scholz (1999a)
*A. cameliae* Hernández-Orts, Georgieva, Landete et Scholz, 2019	Argentina*	19–24	Hernández-Orts et al. (2019)
*A. bulbosa*Ukoli, 1968	Ghana*	16	Ukoli (1968)
*A. inglei*Hutton and Sogandares-Bernal, 1959	Egypt*	19	Hutton and Sogandares-Bernal (1958), Scholz (1999a)
*A. italica* Alessandrini, 1906	Italy*	18	Ransom (1920), Travassos (1930)
*A. longa*Ransom, 1920^c^	North*, Central & South America, Europe, North Africa, Middle East	16	Ransom (1920), Scholz (1999a)
*A. macrostoma* (Robinson, 1956)	Mexico, USA*	18	Robinson (1956), Scholz et al. (1997a)
*A. micracantha* Coil et Kuntz, 1960	Turkey*	20	Coil and Kuntz (1960)
*A. minuta* (Looss, 1899) d	Egypt*, Italy	18–20	Looss (1899), Parona (1912)
*A. septentrionalis* (van den Broek, 1967)	Netherlands*	16–20	van den Broek (1967)
*A. sinoecum* (Ciurea, 1933)	Romania*	19–22	Ciurea (1933)
***A. trentinii* n. sp.**	Italy*	**27**–**33**	**present paper**
**Species with a single complete circle of spines & additional spines (in italic)**			
*A. angrense*Travassos, 1916	Argentina, Brazil*	18 + *2*	Ostrowski de Núñez (1974, 1993)
*A. diminuta*Stunkard et Haviland, 1924^e^	Argentina, Mexico, USA*	16 + *2*	Ostrowski de Núñez (1993), Scholz et al. (1997a)
*A. nana*Ransom, 1920	Mexico, USA*	16 + *4* (rarely 14–17 + *3–6*)	Font et al. (1984a), Scholz et al. (1997a, 2001)
*A. nunezae* Scholz, Vargas-Vázquez, Vidal-Martínez et Aguirre-Macedo, 1997	Mexico*	24–28 + *6–10*	Scholz et al. (1995, 1997a, b)
*A. pithecophagicola*^f^	Philippines*	16 + *4*	Scholz (1999b)
**Species with a double complete circle of circumoral spines**			
*A. ampullacea* Miller et Harkema, 1962	USA*	20–27 + 20–27	Miller and Harkema (1962), Scholz et al. (2001)
*A. angeloi*Travassos, 1928	Argentina, Brazil*	14 + 14	Ostrowski de Núñez (1998)
*A. branchialis*Timon-David, 1961	France*	16–18 + 16–18	Timon-David (1961)
*A. chandleri*Lumsden, 1963	Mexico, USA*	27–28 + 27–29	Lumsden (1963), Scholz et al. (1997a)
*A. coleostoma* (Looss, 1896)	Egypt*	16 + 16	Looss (1896)
*A. felippei*Travassos, 1929^g^	Argentina, Brazil*, Mexico, Puerto Rico, USA	16 + 16	Scholz et al. (2001), Santos et al. (2007)
*A. gemina* Font, Overstreet et Heard, 1984	Mexico, USA*	27–32 + 28–32	Font et al. (1984b), Scholz et al. (2001)
*A. hadra*Ostrowski de Núñez, 1992	Argentina*	18–21 + 18–21	Ostrowski de Núñez (1992)
*A. leighi*Burton, 1956	Mexico, USA*	24–26 + 24–26	Burton (1956), Scholz et al. (2001)
*A. mcintoshi*Price, 1936	Mexico, USA*	18–21 + 18–21	Price (1936), Scholz et al. (2001)
*A. megalocephala*Price, 1932	Mexico, USA*	36–40 + 36–40	Price (1932), Scholz et al. (2001)
*A. pachycystis* Schroeder et Leigh, 1965	USA*	22–29 + 22–29	Schroeder and Leigh (1965)
*A. paratenuicollis* Nasir, Lemus de Guevara et Díaz, 1970	Venezuela*	11 + 11^h^	Lemus de Guevara et al. (1968), Nasir et al. (1970)
*A. patagoniensis* Hernández-Orts, Montero, Crespo, García, Raga et Aznar, 2012	Argentina*	18–21 + 18–21	Hernández-Orts et al. (2012, 2019)
*A. secunda* Ostrowski de Núñez, 2001	Argentina*	16 + 16 (rarely 15 or 17/row)	Ostrowski de Núñez (2001)
*A. sexidigita* Martin et Steele, 1970	USA*	29–30 + 29–30	Martin and Steele (1970)
*A. tertia* Ostrowski de Núñez, 2001	Argentina*	16 + 16 (rarely 15 or 17/row)	Ostrowski de Núñez (2001)
**Species without circumoral spines**			
*A. pindoramensis*Travassos, 1928	Brazil*	no spines	Travassos (1928), Simões et al. (2006)
*A. plana*Linton, 1928	USA*	allegedly no spines	Linton (1928)
*A. intermedius*Srivastava, 1935	India*	N/A	Srivastava (1935)

a Species are listed in alphabetical order, with taxa possessing a single complete circle of circumoral spines listed first, followed by species with a single complete circle and additional spines on the ventral side, and finally species with two complete circles of spines; *A. pindoramensis* and *A. plana*, which are devoid of circumoral spines, are listed last.

b Country with type locality marked by asterisk.

c Synonyms of *A. longa*: *A*. *arnaldoi*Travassos, 1929, *A*. *byrdi* (Robinson, 1956), *A*. *longicollis* (Kuntz & Chandler, 1956).

d Reported as *Distomum* (*Ascocotyle*) *minutum* in dog by Parona (1912).

e Synonym of *A. diminuta*: *A. lageniformis* Chandler, 1941.

f Type species.

g Synonyms of *A. felippei*: *A. puertoricensis*Price, 1932, *A. tenuicollis* Price, 1935.

h This number is apparently incorrect (underestimated) because lateral spines were not counted – see Fig. 1 in Nasir et al. (1970).

To obtain adults, four 3 days old ducklings (*Anas platyrhynchos domesticus* var. American pekin) were fed with *A. fasciatus* infected with metacercariae of the new species. Fish infection was verified at dissection microscope before feeding the ducklings. Ten fish were chopped together and fed to four ducklings. The fish were previously checked under dissection microscope to verify the presence of metacercariae, always over 100 in number. Ducklings were kept in a ground stand in a facility of the State Veterinary Institute of Lombardia and Emilia-Romagna in Forlì. They were subjected daily to coprological examination until the finding of digenean eggs, i.e., 5 days p.i. Ducklings were then euthanised by cervical dislocation and their intestine was examined to collect the parasites. The adults (several dozens) were then isolated from the gut of experimentally infected ducklings 5 days post-infection, washed in sterile saline and then fixed in 70% ethanol.

### 2.2 Morphological data

Adults were clarified with Amman lactophenol or stained with Mayer's carmine, dehydrated, clarified with eugenol, and mounted in Canada balsam as permanent preparations. Specimens were observed under light microscope Olympus BX 51. Line drawings were made using a drawing tube and measurements were taken using imaging software NIS-Elements (Nikon, Campi Bisenzio, FI, Italy) and QuickPhoto (Olympus, Tokyo, Japan). Measurements are expressed in micrometres (μm) and are presented as the range, with the mean followed by standard deviation (SD); n = number of measurements, usually also corresponding to the number of specimens measured, with few exceptions, such as size of circumoral spines. For scanning electron microscopy (SEM), adults were dehydrated through a graded alcohol series, dried by hexamethyldisilazane, coated with gold and then examined using a Phenom XL G2 Desktop SEM (Thermo Fisher Scientific) operating at 5 kV.

#### 2.2.1 Molecular data

Total DNA, from both a metacercaria and an adult, were extracted using PureLink Genomic DNA Kit (Life Technologies, Carlsbad, California) following the manufacturer's protocol. The 28S rRNA gene was amplified with the primers U178 (forward 5′-GCACCCGCTGAAYTTAAG-3′) and L1642 (reverse 5′-CCAGCGCCATCCATTTTCA-3′) of Lockyer et al. (2003). The thermal cycler program (Tpersonal, Biometra) was a denaturation step at 94 °C for 2 min, 40 cycles of 30 s at 94 °C, 30 s at 52 °C and 2 min at 72 °C and followed by an extended elongation step at 72 °C for 10 min. Amplified products were resolved on 1% agarose gel stained with SYBR Safe DNA Gel Stain in 0.5X TBE (Molecular Probes, Life Technologies). For sequencing, bands were excised and purified by NucleoSpin Gel and PCR Cleanup (Mackerey-Nagel, Düren, Germany) and sequenced with an ABI 3730 DNA analyzer at StarSEQ GmbH (Mainz, Germany).

Consensus sequences were assembled with Vector NTI AdvanceTM 11 software (Thermo Fisher Scientific, Carlsbad, California). The sequences were compared with previously published data by BLAST tools (https://blast.ncbi.nlm.nih.gov/Blast.cgi). Multiple sequence alignments were constructed by ClustalW in BioEdit 7.2.5 (Hall, 1999). Phylogenetic tree and models of nucleotide evolution (Bayesian Information Criterion) were calculated using MEGA version X (Kumar et al., 2018). To infer the evolutionary history Maximum-Likelihood (ML) method based on GTR + G + I model with 1000 replicates were used. The newly generated sequences were aligned with the sequences reported by Hernández-Orts et al. (2019) to build the ML tree. The new sequences are published in GenBank under the following accession numbers MZ654879 (metacercaria) and MZ654880 (adult).

## 3 Results

### 3.1 *Ascocotyle trentinii* n. sp. Fig. 1, Fig. 2

*Type-host*: *Anas platyrhynchos domesticus* (experimental host); natural host of metacercariae: *Aphanius fasciatus*.

*Site of infection*: Small intestine.

*Type-locality*: Cervia salt marshes, Emilia-Romagna Region, Italy (44°15′15.17″N; 12°20′02.57″E), the site where metacercariae, used to experimentally infect the type host, were collected from *Aphanius fasciatus*.

*Other localities*: Santa Gilla lagoon, Sardinia, Italy (Culurgioni et al., 2014 – as *Ascocotyle* sp. 3).

*Type-material*: Holotype and 13 paratypes are deposited at the Helminthological Collection of the Institute of Parasitology, Biology Centre of the Czech Academy of Sciences, České Budějovice, Czech Republic (IPCAS D-846) and the Natural History Museum, Geneva, Switzerland (MHNG-PLAT-0138866 – paratypes; MHNG-PLAT-0138867 – vouchers).

ZooBank number: urn:lsid:zoobank.org:act:A0A8E5E9-86FC-45A1-9DFA-065AB568E533.

*Second intermediate host*: Mediterranean banded killifish, *Aphanius fasciatus* (Cyprinodontiformes: Aphaniidae); infected 29 of 35 killifish, i.e., prevalence of 83%, and intensity of infection reaching up to 100 metacercariae/fish; even most heavily infected fish host were sexually mature.

*Etymology*: the species is named after Professor Massimo Trentini, our dear colleague and friend who participated in the initial study of this parasite and prematurely passed away.

*Representative DNA sequences*: newly generated sequences of 28S rDNA (length of 1,622 bp) of an adult and a metacercaria were identical, which confirms they represent different ontogenetic stages of the same species.

### 3.2 Description of adults (Fig. 1, Fig. 2)

**Fig. 1:**
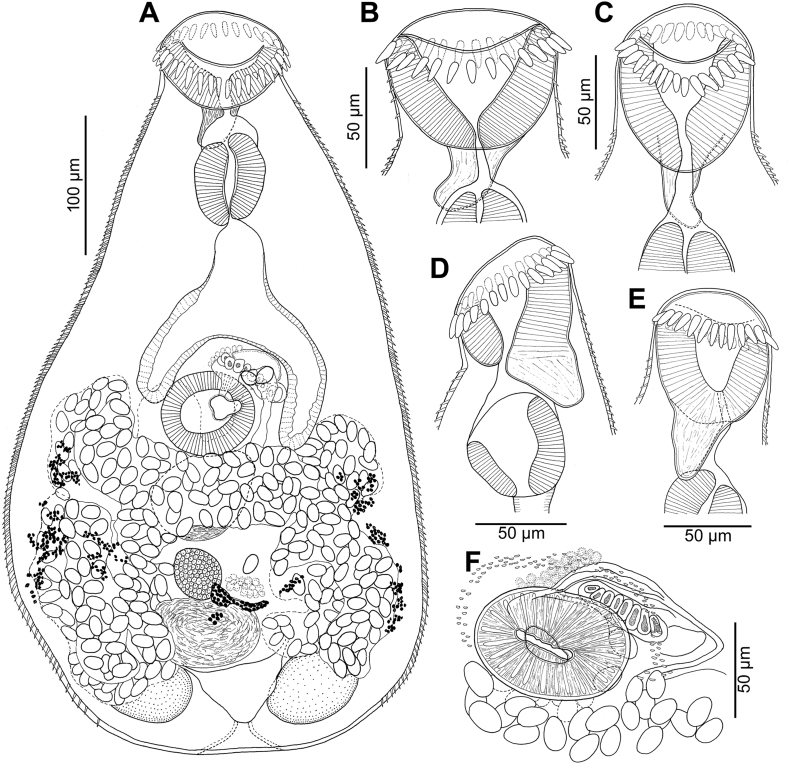
*Ascocotyle* (*Phagicola*) *trentinii* n. sp. from experimentally infected duckling (*Anas platyrhynchos*); holotype (IPCAS D-846). A – total view, ventrally; B–E − oral sucker with one row of circumoral spines and posterior prolongation; F – ventral sucker with ventrogenital sac, ventrally; note gonotyl with 8 refractile pockets.

**Fig. 2:**
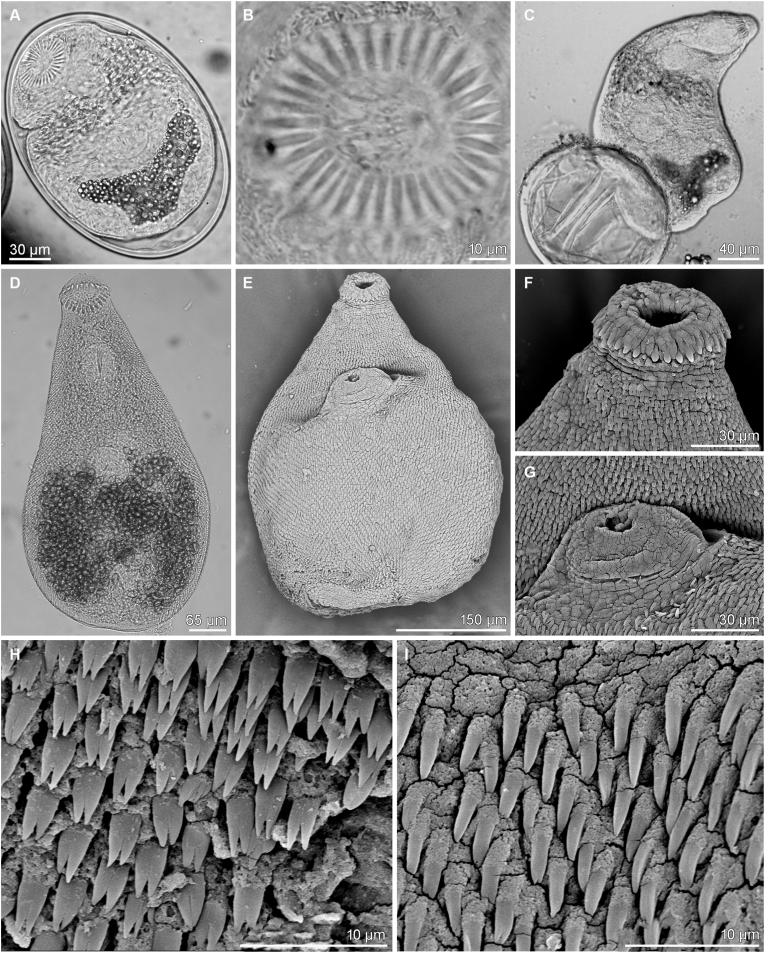
*Ascocotyle* (*Phagicola*) *trentinii* n. sp. metacercariae from *Aphanius fasciatus*: (A) encysted metacercaria; (B) circumoral spines of metacercaria; (C) excysted metacercaria with emptied cyst. *Ascocotyle* (*Phagicola*) *trentinii* n. sp. adults from experimentally infected duckling (*Anas platyrhynchos*): (D) total view, ventrally; light microscopy and (E) Scanning Electron Microscopy (SEM); (F) SEM details of oral sucker with circumoral spines and (G) of ventral sucker; SEM details of (H) bifid tegumental spines and (I) single pointed spines at the bases of both suckers.

Description (based on 29 mounted specimens and 2 specimens studied using SEM): Body pyriform, 440–635 (566 ± 57; n = 20) long and 290–470 (365 ± 43; n = 20) wide, with maximum width at mid-hindbody or slightly more anterior (Fig. 2D, E). Body densely covered with tegumental spines except for region posterior to circumoral spines (Fig. 2F), around opening of ventrogenital sac (Fig. 2G) and posterior extremity (Fig. 1, Fig. 2D, E). Anterior (preacetabular) part of body covered with flat, long and narrow spines with two short, tooth-like projections, 3.5–5.3 (4.2 ± 0.4; n = 20) long and 1.6–2.5 wide (2.0 ± 0.2; n = 20) (Fig. 2H). Posterior (postacetabular) part of body with simple spines with sharp terminal point (tip), 4.4–5.9 (5.1 ± 0.4; n = 20) long and 1.3–1.5 (1.4 ± 0.1; n = 20) wide (Fig. 2I).

Oral sucker subterminal, 58–85 (72 ± 8; n = 27) long and 64–100 (85 ± 9; n = 27) wide, with conical, short posterior appendage, 37–45 (41 ± 6; n = 2) long, reaching anterior margin of pharynx posteriorly. Oral sucker surrounded by single row of 29–33 (30 ± 1) massive, spearhead-like circumoral spines (Fig. 1, Fig. 2F); spines 12–18 (14.5 ± 1; n = 50) long and 3–6 (4.5 ± 1; n = 50) wide (in frontal view). Prepharynx short 6–26 (15 ± 7; n = 6) long, but distinct; pharynx widely elongate, strongly muscular, 52–85 (72 ± 8; n = 29) long and 42–80 (65 ± 11; n = 29) wide (Fig. 1, Fig. 2D). Oesophagus short, 29–67 (47 ± 15; n = 6) long; intestinal caeca 114–170 (143 ± 18; n = 7) long and 18–42 (31 ± 8; n = 7) wide, short, reaching posteriorly only level of posterior margin of ventral sucker. Ventral sucker well-developed, deeply embedded, spherical to subspherical, wider than long, 54–79 (67 ± 7; n = 26) long and 60–85 (77 ± 7; n = 26) wide, nearly equatorial (slightly dextral), situated at 43–49% (45% ± 3%; n = 5) of body length from anterior extremity. Opening of ventral sucker transversely oval to widely oval; ratio of width of suckers (OS *vs* VS) 1: 0.94–1.33 (1.11 ± 0.12).

Testes double, unlobed, widely oval to subspherical, symmetrical, situated close to posterior margin of body, lateral to excretory bladder, 43–107 (75 ± 20; n = 7) long and 37–74 (57 ± 14; n = 7) wide (dextral testis) and 58–78 (75 ± 13; n = 7) long and 48–78 (60 ± 9; n = 7) wide (sinistral testis) (Fig. 1A). Seminal vesicle voluminous 59–92 (75 ± 12; n = 8) long and 42–72 (58 ± 9; n = 8) wide, difficult to observe because of numerous overlaying eggs, posterior to ventral sucker. Ventrogenital sac formed, containing obliquely situated pad-like gonotyl 12–22 (17 ± 4; n = 7) long and 43–53 (48 ± 4; n = 7) wide, composed of about 7–9, usually 8 refractile bodies. Opening of ventrogenital sac narrow, transverse, slit-like, slightly sinistral to longitudinal body axis.

Ovary spherical 34–55 (41 ± 6; n = 9) long and 32–69 (49 ± 15; n = 9) wide, median, at level of testes. Seminal receptacle 43–74 (59 ± 11; n = 12) long and 39–104 (79 ± 11; n = 12) wide, voluminous, transversely oval, median, posterior to ovary. Laurer's canal not observed. Vitellarium formed by irregularly-shaped follicles grouped in two posterolateral groups between posterior margin of ventral sucker to posterior half of testes. Common vitelline ducts slightly sinuous, ventrally passing over anterior part of seminal receptacle, medially joined and widened to form vitelline reservoir situated posterolateral to ovary.

Uterus tubular, forming numerous loops between anterior margin of ventral sucker and posterior margin of testes, reaching slightly posterior to testes. Metraterm opening sinistrally into ventrogenital sinus. Eggs thick-walled, operculate, 17–21 (19 ± 1; n = 20) long and 11–12 (11.5 ± 0.5; n = 20) wide. Excretory vesicle V-shaped, with short and wide stem.

SEM observation of adults revealed the presence of tegumental spines with different shape: bifid in almost whole-body surface (Fig. 2H), with some spines characterised by two main tips plus a small and less developed median tip randomly present. The last type, slightly surrounding the base of both suckers, shows a single sharp apex (Fig. 2I).

### 3.3 Description of metacercariae (Fig. 2 A–C)

Description (based on 20 live metacercariae from the visceral serosa of *Aphanius fasciatus*). Encysted metacercariae oval, 173–250 long (213 ± 26) and 140–160 (154 ± 8). Cyst wall double, with hyaline, elastic outer wall 3.5 μm thick and transparent membraneous inner layer. Excysted metacercariae pyriform, covered with tiny tegumental spines. Oral sucker bearing 27–33 (30 ± 2; n = 11) circumoral spines and muscular posterior appendage (posterior prolongation). Intestinal caeca reaching posteriorly to level of ventral sucker; they do not contain spherical discs. Region between pharynx and intestinal bifurcation filled with large concentration of gland cells with darker content. Primordia of genital organs (ovary and symmetrical testes) well-developed; primordium of gonotyl visible in some specimens. Excretory vesicle Y-shaped, with short and wide stem, filled with dark excretory granules.

### 3.4 Differential diagnosis

**Fig. 3:**
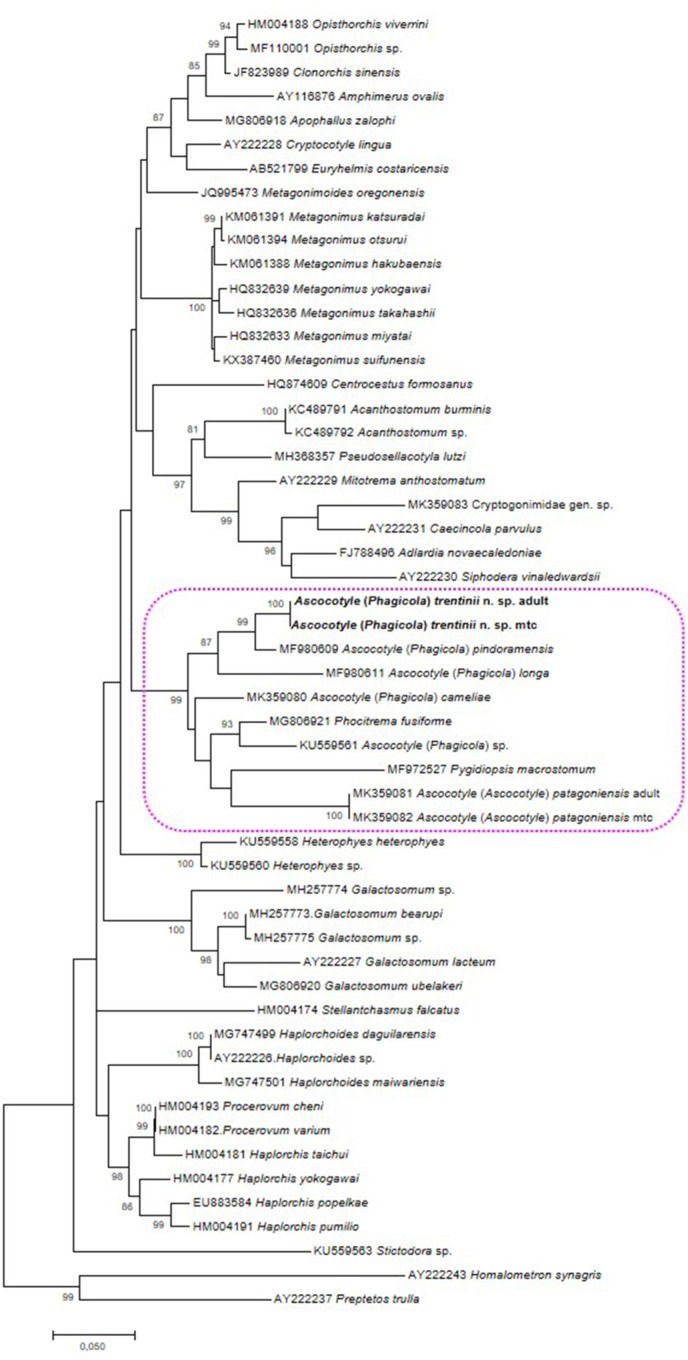
Maximum-Likelihood tree based on the partial sequences of 28S rDNA (including D1-D3 domain) of the Opisthorchioidea. The tree is drawn to scale, with branch lengths measured in the number of substitutions per site. The clade containing species of *Ascocotyle* highlighted. Abbreviations: mtc, metacercariae.

The new species belongs to *Ascocotyle* because it possesses a single row of circumoral spines, vitelline follicles confined to the region between the ventral sucker and testes, and uterine loops not reaching anterior to the ventral sucker. It is placed to the subgenus *Phagicola* Faust, 1920 based on the above-mentioned characteristics, but Pearson (2008) did not recognize individual subgenera of *Ascocotyle* (and molecular data support this conclusion; see also Fig. 3 in the present paper).

*Ascocotyle* (*Phagicola*) *trentinii* n. sp. differs from the other members of this subgenus as well as other species of *Ascocotyle* by the number (29–33) of circumoral spines 12–18 μm long and 3–6 μm wide (see Table 1 for the number of spines in all species of *Ascocotyle*), and by the morphology of the gonotyl, which is composed of about 8 large refractile pockets.

The tegumental spination pattern of the new species observed by SEM differs from that described in other congeneric species that were studied using SEM. The most recently described species, *A.* (*P*.) *cameliae* Hernández-Orts, Georgieva, Landete et Scholz, 2019 from the Magellanic penguin, *Spheniscus magellanicus* (Forster), in Argentina, possesses pectinate tegumental spines, but the number of their terminal points decreases continuously from three on the anterior third of the body to a single point in the posterior part of body surface (Hernández-Orts et al., 2019). *Ascocotyle* (*P*.) *pindoramensis* shows digitiform tegumental spines and is devoid of circumoral spines (Simões et al., 2006). Despite these morphological differences, *A.* (*P*.) *pindoramensis* is the sister taxa of *A.* (*P*.) *trentinii* n. sp. as showed in the ML tree of the 28S rDNA. Both these species are reported in cyprinodontiform fish, *Poecilia vivipara* Bloch et Schneider and *Aphanius fasciatus*, respectively, and live in similar habitats (brackish waters), but in different continents (South America *versus* Europe).

### 3.5 Remarks

Culurgioni et al. (2014) reported unidentified metacercariae of *Ascocotyle* as *Ascocotyle* (*Phagicola*) sp. 3 from *A. fasciatus* sampled in Santa Gilla lagoon in Sardinia, Italy. Because of a similar number of circumoral spines (>30) and their arrangement in a single row, it is likely that the specimens were conspecific with *A.* (*P.*) *trentinii* n. sp.

### 3.6 Molecular data

The ML tree showed the new species forming a well-supported cluster within the *Ascocotyle* clade (including species placed in the subgenus *Phagicola*), with *A.* (*P.*) *pindoramensis* (0.036 distance, no circumoral spines) as its sister taxon and *A.* (*P.*) *longa* (0.080 distance, a single row of circumoral spines) basal to both species (Fig. 3).

## 4 Discussion

*Ascocotyle* (*P.*) *trentinii* n. sp. is the first new species of *Ascocotyle* described from Europe after more than a half century. This may be caused by little attention paid to search for these tiny trematodes, which mature in fish-eating birds; these host are usually protected in most European countries and thus difficult to examine for parasites. Another reason may be relatively rare occurrence of heterophyids in Europe and their low species diversity in temperate zones, especially compared with that in subtropical and tropical regions (Scholz et al., 1997a, b; 2001).

Molecular phylogenetic analysis placed the new species within a well-supported clade composed from all but two species of *Ascocotyle* (Fig. 3). The only species that do not belong to *Ascocotyle*, thus making this genus non-monophyletic, are *Phocitrema fusiforme* Goto et Ozaki, 1930 from seals, sea otter and Arctic fox in northern Pacific, and *Pygidiopsis macrostoma* Travassos, 1928, a poorly known species originally described from a single specimen from *Rattus norvegicus* (Erxleben) in Brazil. In fact, both species are morphologically similar to those of *Ascocotyle* (including distribution of internal organs and structure of the gonotyl with pockets – see Pearson, 2008), the only difference being in the absence, rather than presence, of a posterior, tapering appendage of the oral sucker. It is obvious that validity of *Phocitrema* Goto et Ozaki, 1930 and *Pygidiopsis* Looss, 1907 should be critically revised, based on a broader dataset of sequenced taxa of all genera. Hernández-Orts et al. (2019) also found *P. fusiforme* to be closely related to species of *Ascocotyle* (see Fig. 6 in that paper).

The new species completes its life cycle in brackish waters as the fish second intermediate host occurs mainly in coastal lagoons and tolerates high salinity (Leonardos et al., 1996). The recent discovery of *A. trentinii* n. sp. is somewhat surprising considering extraordinarily high prevalence and intensity (83%, intensity of infection up to 100 metacercariae) of its infection in the Mediterranean banded killifish in Italy. This cyprinodontiform fish is a short-lived, non-commercial species widely distributed in the Mediterranean basin (Bertoli et al., 2019). It lives in saltworks, brackish habitats and lagoons with high salinity levels (up to 65‰) and tolerating a wide range of temperature variation, between 4 and 40 °C (Leonardos and Sinis, 1998; Leonardos, 2008). Due to the high relevance of brackish environments as biodiversity hot spots and buffer areas against extreme and adverse weather events, *A. fasciatus* is considered as an ‘umbrella species’ for these environments (Simberloff, 1998; Roberge and Angelstam, 2004; Valdesalici et al., 2015; Bertoli et al., 2019) and, is listed among the “Least Concern” species in the IUCN Red List (Rondinini et al., 2013) and is reported in the Annex II of the European Habitat Directive (92/43/CEE). This fish is also tested for biological control of mosquito larvae in a brackish area in northern Italy (Salines of Cervia) and the results on controlled reproduction seem to be very promising (Mordenti et al., 2012).

Since *A. fasciatus* is widely distributed in the Mediterranean region, one would expect that metacercariae of the new trematode would be found earlier during surveys on fish parasites in coastal lagoons. The absence of focus of fish parasitologists on this tiny fish may be one of most plausible explications why *A. trentinii* n. sp. was not described earlier. Another possible reason could be restricted distribution of the new species to small areas in Italy. However, an unpublished observation of metacercariae of *A. trentinii* n. sp. in *Aphanius fasciatus* from Sardinia does not support this assumption about endemicity of the new trematode in Italy (J. Culurgioni, personal communication).

The natural definitive host of *A. trentinii* n. sp. is still unknown. The Cervia salt marshes, where the Mediterranean banded killifish were sampled, are characterised by a very rich fauna of fish-eating birds, such as several species of the Ardeidae, Laridae and Anatidae, which may serve as possible definitive hosts of heterophyid trematodes, including the new species. Especially birds of the families Ardeidae are common definitive hosts of trematodes linked to aquatic environments that include fish.

However, most lagoons are protected areas and it is difficult to examine fish-eating birds to obtain adult worms in natural definitive hosts, to better catalogue the biodiversity and to understand better ecological connections in this peculiar ecosystem. The Cervia salt pans host more than 30 sedentary species of birds (more than 100 species in total), among which herons (*Ardea cinerea*, *Ardea alba*, *Egretta garzetta*), larids (*Chroicocephalus ridibundus*) and anatids (*Anas platyrhynchos*, *Spatula clypeata*, *Mareca penelope*, *Tadorna tadorna*). Since several years there is also a stable population of flamingos (*Phoenicopterus roseus*). Considering the eating behaviour of the above mentioned species, the herons seem the most promising potential definitive hosts of *A. trentinii*. However, they live in a protected area, which makes their parasitological examination hardly possible.

Some non-invasive methods such as eDNA screening might help to detect foci of the parasite transmission by detecting its DNA from the eggs, miracidia or cercariae in water samples. Monitoring of the local malacofauna might also help to identify the natural first intermediate host of the new species.

## Declaration of competing interest

There is no conflict of interest in the submitted manuscript entitled “A new species of *Ascocotyle* Looss, 1899 (Digenea: Heterophyidae): the first species newly described from Europe after more than half a century.”
